# Does Inconsistent Social Support Matter? The Effects of Social Support on Work Absorption Through Relaxation at Work

**DOI:** 10.3389/fpsyg.2020.555501

**Published:** 2020-12-03

**Authors:** Shan Xu, Youxin Zhang, Bingran Zhang, Tao Qing, Jiafei Jin

**Affiliations:** ^1^School of Business Administration, Southwestern University of Finance and Economics, Chengdu, China; ^2^School of Management, Harbin Institute of Technology, Harbin, China

**Keywords:** family-supportive supervisor behavior, family support, relaxation at work, work absorption, daily shift

## Abstract

Drawing upon the conservation of resources theory and social exchange theory, we examined the effects of family supportive supervisor behavior (FSSB) and family support (FS) on work absorption at the within- and between-person levels. A 10-day study of 91 workers using 710 observations was employed. At the within-person level, the results suggested that daily relaxation at work mediated the relationships between daily FS, daily shifts in FS, and daily work absorption. However, at the between-person level, the results revealed that chronic relaxation at work mediated the relation between the average level of FSSB/FS and chronic work absorption. We conclude that FSSB/FS plays a vital role in relaxation at work and work absorption at the within- and between-person levels.

## Introduction

Over the past two decades, a burgeoning body of research has examined the benefits of social support (e.g., work support and family support) for employees (Adams et al., 1996; Russo et al., 2015). For example, researchers have found that different types of social support can buffer the negative effects of workload and relieve work–family conflict (Kossek et al., 2011). Among the various types of social support, we seek to understand how family oriented support affects employees’ workplace activities and focus specifically on family supportive supervisor behavior (FSSB) and family support (FS). This is because work and life compete for an individual’s limited resources and energy, and family oriented support is important for employees who are coping with family demands while concentrating on their work (Jin et al., 2014). According to Shockley and Allen (2013), rather than considering support in general, it is important to examine how support in a specific domain (e.g., the family domain) improves individuals’ work activities. French et al. (2018) reviewed two related types of family oriented support, one from the work domain and the other from the non-work domain: FSSB—socially based support provided by supervisors and intended to help employees fulfill family responsibilities—and FS—support provided by family members, which includes emotional support in the form of encouragement or understanding that can aid employee to meet the work demands, and instrumental assistance that can relieve employees’ home-related demands (Lapierre and Allen, 2006).

Most existing research has used conservation of resources (COR) theory to explain how social support benefits employees. However, Halbesleben et al. (2014) proposed that COR theory is dynamic because FSSB and FS have the potential to vary from day to day considerably more than other types of social support do, such as organizational support (e.g., Pluut et al., 2018). A recent daily diary study conducted by Pluut et al. (2018) argued that social support should be conceptualized as a “volatile” resource because individuals who receive this resource experience daily variance. This means that individuals receive fluctuating social support in daily interactions with others such as supervisors, coworkers, and spouses (e.g., ten Brummelhuis and Bakker, 2012). Thus, it is necessary to examine resource gains on a daily basis. Although the “main effects model” of social support examines the antecedent role of social support, it fails to explain how family oriented social support improves positive workplace outcomes, such as work absorption, at the within-person level. Work absorption, defined as the central psychological dimension of work engagement (Rothbard, 2001; Schaufeli and Bakker, 2004), describes an employee’s psychological state, concentration, and immersion while doing his or her work (Dumas and Perry-Smith, 2018). According to COR theory, individuals tend to protect their current resources while building new resources (Halbesleben et al., 2014); thus, we expected that daily FSSB and FS could stimulate the “resource gain” process. Fritz and Sonnentag (2006) argued that relaxation at work could be seen as a resource-providing experience and regarded as an active form of recovery that occurs when employees engage in activities such as taking short naps, stretching, or listening to music during lunch and micro-breaks. We expected that family oriented social support could promote individual relaxation during everyday work breaks and facilitate recovery from daily energy depletion to build new resources, which further helps employees engage in their daily work.

In addition, although these studies have provided convincing evidence that social support exhibits day-to-day variance, little research has considered regarding how ***changes*** between two adjacent days in family oriented social support can affect an employee’s attitudes and behaviors. Wang et al. (2019) argued that social interactions between individuals are relatively episodic and easily influenced by fluctuating characteristics, such as the emotion and energy levels of others (e.g., supervisors, coworkers, and spouses). Imagine that an employee receives less support from his/her supervisors 1 day and more the next; compare this employee with someone who receives consistent support from his/her supervisors. Would the two employees react differently in these two situations? We defined a daily shift in family oriented social support as the relative difference in family oriented social support received between two successive days (Wang et al., 2019). We argue that, rather than simply examining the daily level of social support, shifts in social support must also be considered, as they directly reflect deviations when comparing social support received on 1 day with that received on the previous day within an individual. Comparing daily variance to a consistent daily level of family oriented social support, we argue the following: Based on social exchange theory, the shift in FSSB and FS, which indicates inconsistent support from 1 day to the next, may be harmful for employees. Because upshifts in family oriented social support are more likely to lead to employees sacrificing their break time and relaxing less at work, their daily work absorption is also negatively influenced. By contrast, downshifts in family oriented social support mean that employees receive declining support and need to bear work and family responsibilities by themselves. They have to “catch their breath” and leave work behind during breaks so that they can be absorbed in their daily work.

Additionally, the relationships between variables may not be homogeneous within and across individuals (Hamaker et al., 2007) because the factors that influence covariance may not be relevant across the within- and between-person levels (Brose et al., 2015). In fact, the relationships at the within- and between-person levels differ in not only strength but also direction. For example, Prem et al. (2018) found that challenge appraisal reduced self-regulation effort and hindrance appraisal increased self-regulation effort at the within-person level, whereas neither challenge nor hindrance appraisal could predict self-regulation effort at the between-person level. To address this issue, we also test the chronic effects of FSSB/FS at the between-person level. We define “chronic effects” at the between-person level in relation to the average level of family oriented support experienced over a longer period. Analysis at the between-person level emphasizes that the more FSSB/FS employees receive, the more chronic relaxation at work and chronic work absorption they perceive. The analysis of both within- and between-person effects enables us to specify the respective contributions of FSSB and FS to work absorption.

**FIGURE 1:**
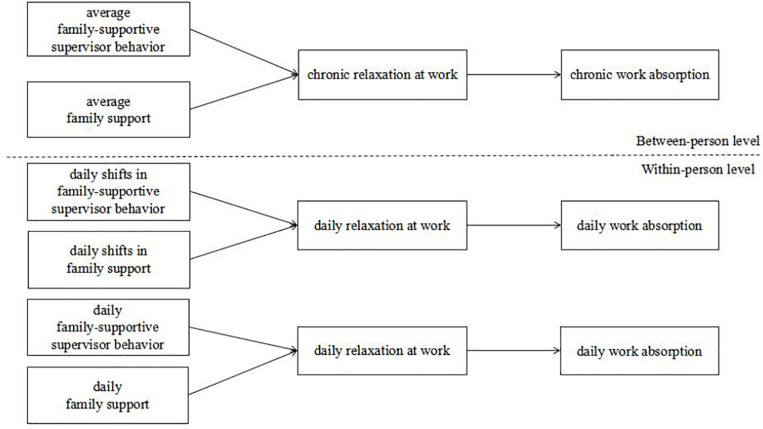
The hypothesized model.

This study contributes to the existing literature on social support in several ways. First, this is the first study to examine the “daily shift” in family oriented social support. We primarily aim to extend the theory and research on social support by introducing the concept of the daily shift in social support, which captures the differences in social support between two adjacent days. Second, we integrated the within- and between-person levels to thoroughly investigate how family oriented social support affects work absorption. Third, following COR theory and social exchange theory, at each level, we tested the indirect effects of social support on work absorption via relaxation at work during the workday. Specifically, we examined the mediating effects of daily relaxation at work on the relationships between daily FSSB/FS, their shifts, and daily work absorption at the within-person level. In addition, we examined the mediating role of chronic relaxation at work on the relationships between the average level of FSSB/FS and chronic work absorption. Therefore, this study provides a fresh perspective on how various types of family oriented support affect employees’ daily workplace activities via a multilevel model. The research model of this study is shown in Figure 1.

## Theoretical Background and Hypotheses

### Daily FSSB, FS, and Daily Relaxation at Work (Within-Person Level)

Family supportive supervisor behavior includes emotional support, role modeling, instrumental support, and creative work–family management from supervisors who are supportive of employees’ family responsibilities (Hammer et al., 2009). FS has been defined as support from family members and includes emotional sustenance and instrumental assistance, which help employees cope with family related demands (King et al., 1995). Until now, most studies on FSSB and FS have been cross-sectional, and these concepts have been treated as relatively stable perceptions. In fact, family oriented social support may derive not only from a relatively stable organizational culture and personality (Bowling et al., 2005; Straub, 2012) but also from social interactions with significant others and episodic events occurring on a daily basis. We propose that on a day-to-day level, employees and family members might experience diverse negative or positive episodes across work and non-work domains. This might cause variance in the emotional support received from supervisors and family members, such as affection and sympathy (Thoits, 1982), as well as instrumental support in the form of task instruction and task assistance (Deelstra et al., 2003). Therefore, FSSB and FS, as important forms of family oriented social support, have day-to-day fluctuations.

Under COR theory, Hobfoll (2002) defined resources as things that “either are centrally valued in their own right or act as a means to obtain centrally valued ends (e.g., social support)” (p. 307) and that are vital for coping with stress. Odle-Dusseau et al. (2012) proposed that “Because organizational work–family resources are commonly implemented in response to employees’ desires and values” (p. 29), FSSB and FS are resources according to Hobfoll’s definition. In addition, one principle of COR theory indicates that “people must invest resources to gain resources and protect themselves from losing resources or to recover from resource loss” (Halbesleben et al., 2014, p. 1338). Thus, we argue that individuals with more FSSB and FS are less likely to worry about family affairs at work and can optionally choose the time and intensity of their relaxation at work and thus recover from resource loss (energy loss). Trougakos et al. (2014) demonstrated that relaxing activities are effective in reestablishing the resources required to decrease work-related fatigue. Therefore, relaxation can be regarded as an energy- and resource-rebuilding process (Halbesleben et al., 2014). More specifically, we predict that employees with high daily FSSB are likely to acquire both job control and autonomy through communicating with their supervisors (Hammer et al., 2009), which can allow them to decide on the time and frequency of their relaxation at work. Simultaneously, supervisors who provide more FSSB understand their subordinates’ struggles regarding work and family demands. This results in the provision of more daily emotional support, such as kind suggestions, or daily instrumental support, such as flexible work arrangements, so that employees are not burdened with family worries during the workday. To our knowledge, non-work demands may also hinder relaxation at work because of their unpredictable and obligatory nature (Demerouti et al., 2009). However, if employees are equipped with high daily FS, their family members can help them take care of necessary matters. Thus, we argue that contextual resources such as FSSB and FS help employees engage in more relaxation at work:

H1: At the within-person level, daily FSSB and FS are positively associated with daily relaxation at work.

### Daily Relaxation at Work and Daily Work Absorption (Within-Person Level)

When the concept of work engagement was introduced, Kahn (1990) regarded it as a dynamic state that fluctuated in response to the external environment. Work absorption is considered to be an important dimension of work engagement, and an absorbed person is characterized by complete concentration and immersion in work, resulting in them feeling that time flies (Dumas and Perry-Smith, 2018). There is evidence that absorption varies across the workday. First, researchers have found that absorption has more daily variance than other dimensions of work engagement (Sonnentag et al., 2010). One study showed that 62.28% of the total variance in absorption was explained at the within-person level (Ouweneel et al., 2012). Second, absorption at work is influenced by many changeable variables, such as positive affect, social and individual resources (Christian and Slaughter, 2007; Rodríguez-Sánchez et al., 2011; van Woerkom et al., 2016).

Many studies have proven that after-work recovery helps employees to be more engaged in their work (Sonnentag, 2003; Sonnentag and Fritz, 2015). However, whether the internal recovery that occurs within a work context (Geurts and Sonnentag, 2006), such as relaxation at work, benefits work-related outcomes still needs to be explored. This is surprising because recovery is a process that may occur throughout the day (Trougakos et al., 2014). Some studies have found that job and personal resources make an individual more engaged (Xanthopoulou et al., 2009). Therefore, we argue that daily relaxation at work can positively predict daily work absorption. Based on COR theory, daily relaxation at work, as a resource-rebuilding process, helps employees compensate for resource loss while coping with work demands. It is beneficial for acquiring the psychological resources needed for task concentration and immersion. Some evidence suggests that daily relaxation activities may not only reduce the impact of work demands on negative affect (Kim et al., 2016) but also directly increase positive affect (Kim et al., 2018). Furthermore, positive affect can motivate people to engage in positive behaviors in the form of work absorption (Trougakos and Hideg, 2009). Thus, we hypothesize the following:

H2: At the within-person level, daily relaxation at work is positively associated with daily work absorption.

According to the COR theory’s corollary, “individuals with resources are better positioned for resource gain” (Halbesleben et al., 2014, p. 1338). As mentioned earlier, FSSB and FS help employees to relax at work because employees do not need to worry about their family obligations. For instance, a person who receives more daily FSSB and FS can have more resources, such as autonomy, to decide on their relaxation time and preferred activities, which is important for daily relaxation at work (Trougakos et al., 2014). In addition, such employees might not need to receive calls from their family members during breaks. High levels of relaxation at work could help employees recover from work depletion and gain more energy to be engrossed in their subsequent tasks. Resources recovered through daily relaxation at work, such as energy, enable employees to be vigorously engaged and fully absorbed in their work. However, a person who receives less daily FSSB and FS has to manage their family problems on their own, making it difficult to relax at work. Poor daily relaxation at work results in continuous resource consumption, which could decrease daily work absorption. Thus, we hypothesize:

H3: At the within-person level, daily relaxation at work mediates the relationships between daily FSSB, FS, and daily work absorption.

### Daily Shift in FSSB/FS, Daily Relaxation at Work, and Daily Work Absorption (Within-Person Level)

Although it is vital to examine the relationship between daily FSSB and FS over time within a person, it is equally important to explore the relative difference between FSSB and FS on a specific day and the previous day, which can be defined as the daily shift in family oriented social support. This is because supervisor and spouse behaviors are easily influenced by personal factors, such as personal emotion or work stress, that can fluctuate (Loi et al., 2009; Straub, 2012). For example, Yang et al. (2016) found that organizational citizenship behavior was predicted by a combination of an increase in positive affect on the next day compared to the day before and a decrease in negative affect on the next day compared to the previous day. In addition, Wang et al. (2019) found that the directionality of the daily shift in interpersonal justice has positive effects on daily psychological detachment. Thus, an “upshift” in interpersonal justice, which can be defined as an increase in employee-perceived interpersonal justice relative to that on the previous day, can increase an individual’s psychological detachment. However, a “downshift” (decrease in employee-perceived interpersonal justice) can decrease an individual’s psychological detachment. Several existing findings support the notion that changes in employees’ affective-related experiences (e.g., justice and support) in the workplace have significant effects on their well-being and behaviors (e.g., Lind, 2001). We argue that employees’ daily relaxation at work is a function of not only daily family oriented support but also changes in such support between two successive days.

Based on Bledow et al. (2013), we used the residual score change across every two consecutive days to measure the daily shift in FSSB and FS. Specifically, the residual score was generated by regressing day t support on day t-1 support. We argue that the daily shift in the two types of family oriented support predicts the daily level of relaxation at work. Specifically, episodic upshifts in FSSB/FS negatively predict daily relaxation at work, but episodic downshifts in FSSB/FS positively predict daily relaxation at work. According to social exchange theory, after obtaining resources, individuals establish high-quality exchange relationships and are obliged to engage in reciprocal behaviors (Settoon et al., 1996). Thus, upshifts in support (increased support from a supervisor and family members), compared to the previous day, determine an employee’s gratitude, which manifests in working instead of relaxing during breaks, thus leading to less relaxation at work. However, less relaxation may mean less recovery from work depletion, which may decrease individuals’ daily work absorption. Conversely, episodic downshifts in FSSB/FS (decreased support from a supervisor and family members) mean that employees need to bear most of their work and family burdens alone, potentially leading to the need to “catch their breath” and distance themselves from work during breaks. Thus, their daily relaxation at work necessarily increases so that they can recover from their workload and find the energy to accomplish their work tasks that day. Thus, we propose the following:

H4: At the within-person level, the directionality of the daily shift in FSSB/FS is negatively associated with daily relaxation at work such that daily upshifts in FSSB/FS negatively predict daily relaxation at work and daily downshifts in FSSB/FS positively predict daily relaxation at work.
H5: At the within-person level, daily relaxation at work plays a mediating role in the relationships between the directionality of the daily shift in FSSB/FS, and daily work absorption.

### Average Level of FSSB, FS, Chronic Relaxation at Work, and Chronic Work Absorption (Between-Person Level)

Although the episodic effect of family oriented social support is important for daily relaxation and daily work absorption, research has found that conclusions drawn from evidence obtained at the within-person level cannot be generalized to the between-person level (Molenaar, 2004). It is necessary to comprehensively consider both episodic effects and chronic effects in a multilevel model. We expected that at the between-person level, daily FSSB and FS over time could extend to chronic levels and have significant effects on chronic relaxation at work and chronic work absorption.

We define the average level of FSSB and FS at the between-person level as the mean FSSB and FS that individuals received over a period. Chronic relaxation at work represents the average level of relaxation over a certain period. Therefore, from a chronic perspective, we expected that high average levels of FSSB and FS may facilitate individual chronic relaxation at work. Under high average levels of FSSB, a supervisor may not only understand the employee’s responsibilities at home (Hammer et al., 2013) but also provide them with a flexible work schedule. Similarly, high average levels of FS mean that individuals receive more emotional support and instrumental assistance from family members (King et al., 1995). According to COR theory, high average levels of FSSB and FS over a period could enlarge individuals’ “resource pools,” which help employees achieve a relatively stable state in which they are quite capable of handling work and family responsibilities simultaneously (Ferguson et al., 2016; Crain and Stevens, 2018). This also makes workplace relaxation easier for them. Research has also found that an employee with high average levels of FSSB and FS can avoid feeling trapped by family issues, which therefore do not interfere with their chronic relaxation at work (Muse and Pichler, 2011). Therefore, at the between-person level, high average levels of FSSB and FS are beneficial for chronic relaxation at work. Thus, we propose the following:

H6: At the between-person level, the average levels of FSSB and FS are positively associated with chronic relaxation at work.

Further, based on COR theory, we also predict that chronic relaxation at work can improve chronic work absorption through stable personal resources at the between-person level. Existing research has found that relaxation can enhance individuals’ recovery self-efficacy (Hahn et al., 2011) and increase organization-based self-esteem (Lee et al., 2016). Thus, we expected that chronic work absorption could be enhanced through stable personal resources in the long term, such as through recovery self-efficacy, optimism, and organization-based self-esteem (Xanthopoulou et al., 2007). When employees experience high average levels of relaxation at work over a long period, they are equipped with more psychological resources (e.g., recovery self-efficacy) that enable absorption in their work. As such, we hypothesize the following:

H7: At the between-person level, chronic relaxation at work is positively associated with chronic work absorption.

We predict that chronic relaxation at work plays a mediating role in the relationship between the average level of family oriented social support and chronic work absorption. According to COR theory (Halbesleben et al., 2014), individuals tend to protect current resources and obtain new resources. We argue that high family oriented social support can act as a critical resource that improves an individual’s self-efficacy and ability to handle work and family responsibilities, which can facilitate relaxation at work. In addition, resources such as energy that are rebuilt from relaxation are more likely to allow an employee to be absorbed in his/her work. Therefore, in the long run, we predict that high average levels of FSSB and FS improve employees’ psychological resources, which helps them relax at work and obtain more resources, such as sustainable energy. Such resources could help the employee to be absorbed in his/her work. Hence, we hypothesize:

H8: At the between-person level, chronic relaxation at work mediates the relationships between the average level of FSSB, FS, and chronic work absorption.

## Materials and Methods

### Sample and Procedure

To explore the theme of this study, we used a diary study to measure each variable: FSSB, FS, relaxation at work, and work absorption. Two authors of this study and several graduate students posted research advertisements on social media platforms to recruit qualified participants. In addition, we encouraged participants to share information with others who might be interested in this survey via the snowball sampling method (Biernacki and Waldorf, 1981). We strictly controlled the work background of the participants, and all of them worked for an average of 40 h per week.

Prior to data collection, the researchers conducted training for the participants to explain the purpose of the study and the data collection procedures. After the survey began, two researchers issued questionnaires to the participants at 4:00 p.m. every day and reminded them to recall their day’s FSSB, FS, relaxation at work, and work absorption. We checked their responses at 7:00 p.m. every day to provide feedback and track their answers. We also reminded participants to finish their questionnaires before going to sleep. After collecting demographic information on Sunday, we obtained daily work data (FSSB, FS, relaxation at work, and work absorption) from Monday to Friday over two workweeks. At the end of the last workday, we thanked the participants and distributed a $20 payment to each. Participants who did not complete all the surveys were paid based on the number of surveys completed.

The participants of this study were mainly from Sichuan, Yunnan, Heilongjiang, and Beijing in mainland China. A total of 105 questionnaires were received. Only those individuals who participated for at least 2 days were included in the analysis (Wang et al., 2019). This left 91 valid questionnaires with a total of 710 observations. The data have a nested structure (each participant has data points nested within 10 working days). As our model hypothesized relationships between daily shifts in FSSB/FS (Day t’s FSSB/FS regressed on Day t-1’s FSSB/FS), daily relaxation at work (measured on Day t), and daily work absorption (measured on Day t), the maximum number of useful daily observations provided by each participant was eight (Days 2–5 in each workweek). The participants completed 710 out of a possible total of 728 daily surveys (91 participants × 8 days). Among the 91 valid samples, men accounted for 43.8%, and the average age of the participants was 32.93 years. Slightly more than half (57.5%) of the participants had a bachelor’s degree, the average tenure was 7.18 years, 51.1% of the participants had children, and 75.6% had working spouses.

### Measures

As all of our participants were Chinese, we translated and back-translated all the scales from English to Chinese (Brislin, 1980). To avoid potential inaccuracies as far as possible, we also interviewed several HR managers with rich managerial experience in their respective industries. Cronbach’s alpha was used to measure the internal consistency of each instrument.

#### Daily Family Supportive Supervisor Behavior

We measured FSSB using a four-item scale developed by Hammer et al. (2013). A sample item from this four-item scale is as follows: “Today, my supervisor made me feel comfortable talking to him/her about my conflicts between work and non-work.” The internal consistency reliability of this scale is 0.94.

#### Daily Family Support

Family support was assessed by King et al.’s (1995) four-item scale. A sample item from this measure is “Today, someone in my family took on extra household responsibilities when my job got very demanding.” The internal consistency reliability of this scale is 0.91.

#### Daily Relaxation at Work

To measure relaxation at work, we used the four-item subscale of the Recovery Experience Questionnaire (Sonnentag and Fritz, 2007). A sample item from this measure is “Today, during within-day work breaks, I did relaxing things.” The internal consistency reliability of this scale is 0.91.

#### Daily Work Absorption

Work absorption was measured by the four-item subscale of Rothbard’s (2001) engagement scale. A sample item from this measure is “Today, when I was working, I was totally absorbed by it.” The internal consistency reliability of this scale is 0.76.

#### Control Variables

To rule out the potential confounding effects of demographic variables, gender, age, number of children, whether their spouse works, education, and work years were used as control variables at the between-person level. Considering that psychological detachment is closely related to relaxation at work (Sonnentag and Fritz, 2007), we also used the four-item subscale of the Recovery Experience Questionnaire; a sample item from this scale is “Today, during work breaks, I distanced myself from my work.” Daily psychological detachment at the within-person level and the average level of psychological detachment at the between-person level were also used as control variables.

### Analysis

The data have a nested structure. A total of 10 daily surveys (including eight daily shift assessments, for a total of 710 observations) were nested within each person (91 participants); thus, there were 710 observations in level 1 (within-person level) and 91 observations in level 2 (between-person level). We used Mplus 7 (Muthen and Muthen, 2011) and multilevel path analysis to estimate the multilevel nested structural model in this study.

In accordance with Bledow et al. (2013), we regressed FSSB/FS on the focal day based on FSSB/FS at t-1 (e.g., on the previous day) and saved the residual variance. Thus, we retained the residual variance as the variable of the daily shift in FSSB and FS. We also operationalized the average levels of FSSB, FS, chronic relaxation at work, and chronic work absorption as the means of the daily scores over 10 days.

At the within-person level, we estimated the relationships between the daily shift in FSSB/FS and daily consequences (e.g., daily relaxation at work and daily work absorption). At the between-person level, we estimated the relationships between the average level of FSSB/FS and the average level of the consequences (chronic relaxation at work and chronic work absorption). We entered the control variables in all models.

## Results

We conducted confirmatory factor analysis of the main variables involved in the study (FSSB, FS, relaxation at work, and work absorption). The results showed that the four-factor model proposed in this study fit the data well, χ^2^(*df* = 98) = 262.20, SRMR = 0.04, RMSEA = 0.05, CFI = 0.95, TLI = 0.93. In addition, an alternative three-factor model (combining FSSB and FS into one factor), an alternative two-factor model (combining FSSB and FS into one factor and relaxation at work and work absorption into one factor), and a single-factor model (combining FSSB, FS, relaxation at work, and work absorption into a single factor) were tested. The results showed that, compared to those of the four-factor model proposed in this study, the other model fit indexes were significantly worse (see Table 1). These model comparison results showed that the measures did capture distinct constructs.

**TABLE 1:** Confirmatory factor analysis.

Model	χ^2^	*df*	χ^2^/*df*	SRMR	RMSEA	CFI	TLI
Four-factor model	262.20	98	2.68	0.04	0.05	0.95	0.93
Three-factor model	1001.73	101	9.92	0.15	0.11	0.70	0.64
Two-factor model	1345.62	103	13.06	0.18	0.13	0.58	0.51
One-factor model	2116.02	104	20.35	0.22	0.17	0.32	0.22

*Four-factor model, family supportive supervisor behavior, family support, relaxation at work, work absorption; Three-factor model, family supportive supervisor behavior + family support, relaxation at work, work absorption; Two-factor model, family supportive supervisor behavior + family support, relaxation at work + work absorption; One-factor model, family supportive supervisor behavior + family support + relaxation at work + work absorption.*

The means, standard deviations, and correlations at the within- and between-person levels are presented in Table 2. Before testing the hypotheses, we examined the proportion of variance at the between-person level. As shown in Table 3, the within-person variance for FSSB was 0.28, for FS was 0.22, for relaxation at work was 0.36, and for work absorption was 0.34, whereas the between-person variance for FSSB was 0.57, for FS was 0.30, for relaxation was 0.27, and for work absorption was 0.17. The intra-class correlation coefficient ICC(1) of FSSB was 0.67, of FS was 0.58, of relaxation at work was 0.43, and of work absorption was 0.33. The values for these variables (FSSB, FS, relaxation at work and work absorption) exceeded the value of 0.12 reported by James (1982), suggesting significant variance at the between-person level for these variables. Thus, multilevel modeling was appropriate.

**TABLE 2:** Means, standard deviations, and correlations.

Variables	*M*	*SD*	1	2	3	4	5	6	7	8	9	10	11	12	13	14	15
(1) Gender	1.56	0.49	1														
(2) Age	32.93	7.21	−0.04	1													
(3) Number of children	0.56	0.59	−0.06	0.51**	1												
(4) Spouse works	1.94	0.65	−0.06	0.32**	0.30**	1											
(5) Education	4.36	1.13	0.01	−0.15**	−0.17**	−0.11**	1										
(6) Work years	7.18	7.34	−0.08*	0.85**	0.44**	0.27**	−0.10**	1									
(7) Daily psychological detachment	2.94	0.89	0.03	−0.10**	−0.05	−0.11**	0.10	−0.04	1	0.05	0.08	−0.01	−0.10	0.48**	0.18	0.06	0.10
(8) Daily FSSB	3.28	0.92	−0.05	0.10*	0.02	0.10**	0.09*	0.07	0.02	1	0.21*	0.80**	−0.28**	0.34**	0.19	0.99**	0.22*
(9) Daily FS	3.97	0.72	−0.04	−0.10	−0.09*	0.11**	0.08*	−0.02	0.07	0.23**	1	−0.02	0.42**	0.42**	0.14	0.20	0.99**
(10) Daily shift FSSB	0.00	0.53	−0.03	0.04	0.03	0.05	−0.01	0.03	−0.09*	0.62**	0.02	1	−0.32**	0.14	0.01	0.77**	−0.02
(11) Daily shift FS	0.21	0.43	0.06	−0.07	−0.04	−0.06	0.03	−0.06	−0.11**	−0.07	0.48**	−0.03	1	−0.23*	−0.09	−0.29**	0.39**
(12) Daily relaxation at work	3.53	0.79	−0.15**	0.08*	−0.01	0.06	0.15**	0.03	0.39**	0.23**	0.27**	0.02	−0.14**	1	0.29**	0.36**	0.43**
(13) Daily work absorption	3.14	0.71	0.002	−0.01	−0.003	0.06	−0.02	0.03	0.14**	0.17**	0.14**	−0.02	−0.10**	0.13**	1	0.20	0.16
(14) Average level of FSSB	3.28	0.74														1	0.21*
(15) Average level of FS	3.97	0.55															1

*N*(within-person level) = 710. *N*(between-person level) = 91. FSSB refers to family supportive supervisor behavior. FS refers to family support. Variables 1–7 are control variables. Variables 8–13 are within-person variables. Variables 14 and 15 are between-person variables. Correlations below the diagonal refer to the correlations at the within-person level. Correlations above the diagonal refer to the correlations at the between-person level.*p < 0.05; **p < 0.01.

**TABLE 3:** Parameters estimates and variance components of the null model.

Variables	Intercept b_00_	Within-person variance ($\sigma_{\text{W}}^{2}$)	Between-person variance ($\sigma_{\text{B}}^{2}$)	ICC (1)
Family supportive supervisor behavior	3.28	0.28***	0.57***	0.67
Family support	4.00	0.22***	0.30***	0.58
Relaxation at work	3.54	0.36***	0.27***	0.43
Work absorption	3.15	0.34***	0.17***	0.33

*N*(within-person level) = 710. *N*(between-person level) = 91.b_00_ refers to the grand mean of variables across individuals. The percentage of between-person variance [ICC(1)] is computed through the formula $\sigma_{B}^{2}$/($\sigma_{B}^{2}$ + $\sigma_{W}^{2}$).*p < 0.05; **p < 0.01; ***p < 0.001.

### Hypotheses Test

**FIGURE 2:**
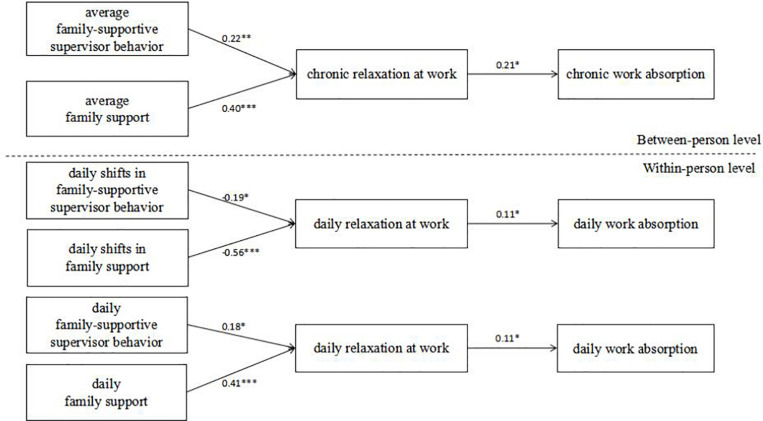
Path model of the results.

Based on Preacher et al. (2010), we used multilevel path analysis to test the hypotheses that simultaneously account for between- and within-person effects. At the within-person level, we tested the effects of daily FSSB/FS on daily relaxation at work and daily work absorption. Further, we tested the effects of the daily shift in FSSB/FS on daily relaxation at work and daily work absorption. In addition, we examined the mediating role of daily relaxation at work in our daily model and daily shift model. At the between-person level, we tested the effects of the average level of FSSB/FS on chronic relaxation at work and chronic work absorption. We also examined the mediating role of chronic relaxation at work. Table 4 and Figure 2 report the coefficients for each key direct path tested in the multilevel model. Table 5 reports the indirect effect coefficients.

**TABLE 4:** Multilevel Model Coefficients for Testing Daily relaxation at work and Daily work absorption.

Predictors	Relaxation at work β(SE)	Work absorption β(SE)
**Between-person level predictors**		
Average level of family supportive supervisor behavior	0.22** (0.08)	
Average level of family support	0.40*** (0.10)	
Chronic relaxation at work		0.21* (0.10)
**Within-person level predictors**		
Daily family-supportive supervisor behavior	0.18* (0.07)	
Daily family support	0.41*** (0.09)	
Daily shift in family-supportive supervisor behavior	−0.19* (0.08)	
Daily shift in family support	−0.56*** (0.10)	
Daily relaxation at work		0.11* (0.06)
Variance explained(between-person)	29%	31%
Variance explained(within-person)	20%	6%

*N*(within-person level) = 710. *N*(between-person level) = 91. Variance explained refers to the percentage of reduction in within-person(or between-person) residual variance after including the predictors. We followed Wang et al. (2019) regarding the formula ($\sigma_{M\,1}^{2}$-$\sigma_{M\,2}^{2}$)/$\sigma_{M\,1}^{2}$ and the calculation of variance explained, where $\sigma_{M\,1}^{2}$ refers to the within-person(or between-person) variance of the null model, and $\sigma_{M\,2}^{2}$ refers to the within-person(or between-person) residual variance of the current model. For example, at the within-person level, the variance of relaxation at work was 0.359 in the null model, whereas the residual variance was 0.286 in the current model, which represented the model including predictors (FSSB and FS), then variance explained of relaxation at work at within-person level was 20% [(0.359-0.286)/0.359 = 0.20]; At the between-person level, the variance of work absorption was 0.167 in the null model, compared with the residual variance of 0.116 in the current model(after adding the relaxation at work into the model), while the explained variance in work absorption at between-person level was 31% [(0.167-0.116)/0.167 = 0.31]. *p < 0.05; **p < 0.01; ***p < 0.001.

**TABLE 5:** Summary of indirect effect coefficients.

Indirect within-person effects	β(SE)
Daily family supportive supervisor behavior → daily relaxation at work → daily work absorption	0.03 (0.02)
Daily family support → daily relaxation at work → daily work absorption	0.05* (0.02)
Daily shift in family supportive supervisor behavior → daily relaxation at work → daily work absorption	0.01 (0.03)
Daily shift in family support → daily relaxation at work → daily work absorption	0.06* (0.03)

**Indirect between-person effects**	**β(SE)**

Average level of family-supportive supervisor behavior → chronic relaxation at work → chronic work absorption	0.03** (0.01)
Average level of family support → chronic relaxation at work → chronic work absorption	0.06*** (0.02)

*N*(within-person level) = 710. *N*(between-person level) = 91.*p < 0.05; **p < 0.01; ***p < 0.001.

Regarding Hypothesis 1, as shown in Table 4 and Figure 2, daily FSSB and daily FS were both positively associated with daily relaxation at work (β = 0.18, *p* < 0.05; β = 0.41, *p* < 0.001) at the within-person level. Thus, Hypothesis 1 was supported. Hypothesis 2 pertains to the relationship between daily relaxation at work and daily work absorption at the within-person level. The results in Table 4 and Figure 2 indicate that daily relaxation at work was positively related to daily work absorption (β = 0.11, *p* < 0.05) at the within-person level, supporting Hypothesis 2.

Hypothesis 3 proposed a mediating role for daily relaxation at work at the within-person level. As shown in Table 5, daily relaxation at work did not mediate the relationship between daily FSSB and daily work absorption (β = 0.03, *ns*.) but did mediate the relationship between daily FS and daily work absorption (β = 0.05, *p* < 0.05). Thus, Hypothesis 3 was partially supported.

To test Hypothesis 4, the relationships between the directionality of the daily shift in FSSB/FS and daily relaxation at work at the within-person level were examined. The results reported in Table 4 and Figure 2 indicate that the directionality of the daily shift in FSSB/FS was negatively related to daily relaxation at work (β = −0.19, *p* < 0.05; β = −0.56, *p* < 0.001). In particular, upshifts in FSSB and FS negatively predicted daily relaxation at work, whereas downshifts in FSSB and FS positively predicted daily relaxation at work. Thus, Hypothesis 4 was supported. Hypothesis 5 proposed a mediating role for daily relaxation at work between the daily shift in FSSB/FS and daily work absorption at the within-person level. The results in Table 5 indicate that daily relaxation at work did not mediate the relationship between the daily shift in FSSB and daily work absorption (β = 0.01, *ns*.) but did mediate the relationship between the daily shift in FS and daily work absorption (β = 0.06, *p* < 0.05). Therefore, Hypothesis 5 was partially supported.

Regarding Hypothesis 6, as shown in Table 4 and Figure 2, the average levels of FSSB and FS were each positively related to chronic relaxation at work (β = 0.22, *p* < 0.01; β = 0.40, *p* < 0.001) at the between-person level. Thus, Hypothesis 6 was supported. Hypothesis 7 pertains to the relationship between chronic relaxation at work and chronic work absorption at the between-person level. The results in Table 4 and Figure 2 show that chronic relaxation at work was positively related to chronic work absorption (β = 0.21, *p* < 0.05) at the between-person level. Thus, Hypothesis 7 was supported. Finally, H8 proposed a mediating role for chronic relaxation at work in the relationships between the average level of FSSB/FS and chronic work absorption at the between-person level. The results in Table 5 show that chronic relaxation at work mediated the relationship between the average level of FSSB and chronic work absorption (β = 0.03, *p* < 0.01) and mediated the relationship between the average level of FS and chronic work absorption (β = 0.06, *p* < 0.001). Thus, Hypothesis 8 was supported.

## Discussion

In this study, we examined relaxation at work as a mediator of an employee’s FSSB, FS, and work absorption at the within- and between-person levels. The findings at the between- and within-person levels were different. At the within-person level, daily FSSB and FS were positively related to daily relaxation at work, but the daily shift in FSSB and FS was negatively related to daily relaxation at work. More importantly, daily relaxation at work mediated only the relationship between FS and daily work absorption but did not mediate the relationship between FSSB and daily work absorption. At the between-person level, in contrast to the mediating role of daily relaxation at work at the within-person level, chronic relaxation at work mediated the relationships between the average level of FSSB/FS and chronic work absorption.

### Theoretical Contributions

This study contributes to existing theory about social support in several respects. First, we contribute a “main effects model” of social support by introducing the concept of the daily shift in social support. Existing research has examined the main effects model and buffering effects model of social support. The main effects model indicated that social support, as a predictor, can directly reduce negative outcomes in the workplace, such as emotional exhaustion and work–family conflict (Lee et al., 2013). The buffering model suggested that social support could moderate the relationships between job demands and an individual’s negative outcomes (Luk and Shaffer, 2005; Seiger and Wiese, 2009). With respect to the main effects model, most existing studies have measured social support as a consistent construct (e.g., Lee et al., 2013). Recently, Pluut et al. (2018) argued that social support should not be treated as a time-invariant construct: day-to-day fluctuations exist because employees may receive more support on one specific day than on other days. However, the main effects model of social support fails to consider the effects of ***changes in family oriented social support*** between a specific day and the day before. We found that although daily FSSB and FS were positively related to daily relaxation at work, the ***daily shift in FSSB/FS*** was negatively related to daily relaxation at work. One possible explanation may be grounded in social exchange theory: compared with a relatively low level of FSSB and FS, high family oriented support facilitates reciprocation by the employee, who then responds to his/her supervisor and family members with a higher time investment and less relaxation at work. This finding is meaningful because it suggests that although family oriented social support is beneficial for employees, *inconsistent* support from supervisors and family members may be harmful for them. It is necessary for supervisors to provide a high level of support, and, more importantly, family support for employees must be consistent. This study responds to calls for further research on the dynamics of the employee-supervisor relationship (Coyle-Shapiro and Shore, 2007) and extends employee-family research by studying daily changes in social support. This is important because it can help researchers and managers understand the time-variant nature of social support. Rather than inconsistent social support from work and non-work domains, consistent family oriented social support is beneficial for individuals’ relaxation at work and work absorption.

Second, Halbesleben et al. (2014) proposed that COR theory is dynamic, and many studies have employed longitudinal designs to account for the fluctuation in resources. However, changes in resources within a workday have been neglected (Xanthopoulou et al., 2009). This study used a diary design to capture the resource gain process. Specifically, we examined the mediating role of daily relaxation at work in the relationships between daily family oriented support (FSSB and FS) and daily work absorption. Our research indicated that daily family oriented social support, as daily family related resources received from supervisors and family members, could stimulate the resource gain process by increasing daily relaxation at work. The recovery process in daily relaxation could help employees gain more resources, such as energy, which can help them to be absorbed in their everyday work.

Third, the existing research has indicated that associations between two variables may not be relevant within and across individuals (Hamaker et al., 2007) because the factors that influence the covariance may vary between these two levels (Brose et al., 2015). Thus, it is more rigorous to consider both between- and within-person variance when investigating the effects of family oriented social support on individuals’ workplace behaviors. The results of our study indicated that at the within-person level, daily relaxation at work mediated only the relationships of daily FS and daily shift in FS with daily work absorption. However, the mediating role of daily relaxation at work in the relationships of daily FSSB and daily shift in FSSB with daily work absorption was not confirmed. These results suggest that daily FS enhanced daily work absorption via daily relaxation at work, but FSSB did not. One possible explanation is that as proximal indicators for workplace behaviors (Seiger and Wiese, 2009), supportive behaviors from family members can directly reduce familial demands and help employees fully concentrate on their work without worrying about family affairs. However, as distance predictors of employees’ workplace behaviors, FSSB can involve emotional support or a flexible work arrangement, but its effects on employees’ relaxation at work and work absorption are long-accumulated and not episodic. Our results for the between-person level supported our conclusion: At the between-person level, the mediating role of chronic relaxation at work in the relationship between FSSB and chronic work absorption was significant. This reminds us that supportive behaviors from family members can help employees relax and concentrate at work in a more direct and timely manner.

### Practical Implications for Managers and Employees

Work absorption is the central component of work engagement and consequently predicts job performance (Halbesleben and Wheeler, 2008). As such, it is important for organizations to explore the antecedents of work absorption, including work and non-work social support and psychological mechanisms. The findings at the between-person level suggest that having multiple sources of support inside and outside the work domain is important for making people more engaged from a long-term perspective. This research provides vital implications for managers.

First, FSSB is considered as a skill and is likely to be promoted through learning (Hammer et al., 2011). Organizations can use computer-based or face-to-face training to enhance supervisors’ FSSB. In addition, depending on the needs of the organization, it may be necessary to evaluate FSSB during the manager recruitment process. For example, structured interview questions on emotional support, role modeling, instrumental support, and creative work–family management can be developed to assess an applicant’s ability in the field.

Second, because of the positive effects of chronic and daily relaxation on work absorption, organizations should teach their employees and managers about the restorative benefits of workday breaks. Employees can adopt various ways to relax, such as progressive muscle relaxation, meditation, and deep breathing, during short breaks (Richardson and Rothstein, 2008) after a period of continuous work. In addition, autonomy over relaxation time is as important as what individuals do during the break (Trougakos et al., 2014). Therefore, supervisors and organizations should attempt to offer activities for relaxation to maximize the benefits of breaks. Another possible intervention is workspace design: Silent rooms, lounges, and green spaces can also be provided for better relaxation.

Third, according to our research results, spouses are the main source of FS, and supportive behaviors from family members are timelier and more direct than those from supervisors. Prior research has demonstrated that spousal support can be increased by encouraging spouses to participate in social events at work (Ferguson et al., 2016). This serves to remind managers that a “family day” for employees’ family members can help these family members understand employees’ work demands and may increase the possibility of family support for employees. However, inconsistent support from families and supervisors makes it difficult for employees to relax and hinders their work absorption. This suggests that employees should communicate with their family members about their roles and responsibilities in advance to avoid sudden demands and interruptions. Organizations should also recognize the vital role that families play and encourage employees to acquire family related resources whenever possible.

### Limitations and Suggestions for Future Research

Our study has several limitations that point to avenues for future research. One limitation is the self-reported data, which are likely to yield common method bias (Podsakoff et al., 2012). Although self-reports may introduce common method bias, we took steps to address this possibility. First, we conducted Harman’s single-factor test. The results showed that common method bias is not a threat in our study because the first factor explained less than 32% of the total amount of variance (Bakker and Xanthopoulou, 2009). In addition, we used the unmeasured latent method factor technique (Podsakoff et al., 2003), in which all items in the model used to measure FSSB, FS, relaxation at work, and work absorption were allowed to load on a common method factor. This latent common method factor was then added to the previously estimated four-factor confirmatory factor analysis model. The results showed that this five-factor model fit the data (χ^2^ = 298.303, *df* = 83, SRMR = 0.05, RMSEA = 0.05, CFI = 0.94, TLI = 0.92) worse than the four-factor model. To control for confounding factors, we recommend that future research employ experimental designs or use multisource data from a broader sample. For example, they could obtain data on the individual’s work absorption from his/her direct supervisors.

Second, we did not find an indirect effect of daily relaxation at work between FSSB and daily work absorption. A more fine-tuned study is needed to explore whether daily FSSB influences daily work absorption through a different mechanism at the within-person level because more variance occurs within one workday. We encourage future research to examine the effects of other mediators (e.g., psychological detachment) on the relationship between FSSB and work absorption at the within-person level. Research has found that cognitive micro-break activities, such as reading a newspaper or making personal plans for the upcoming weekend (Kim et al., 2016), are beneficial for employees’ short-term detachment from work demands. This is critical for employees to recover from their workload and acquire meaningful resources for work absorption.

## Data Availability Statement

The raw data supporting the conclusions of this article will be made available by the authors, without undue reservation.

## Ethics Statement

Written informed consent was obtained from the individual(s) for the publication of any potentially identifiable images or data included in this article.

## Author Contributions

SX, TQ, and JJ conceived the idea of the study, collected the data, and provided a theory guide. SX and YZ wrote the manuscript. SX and BZ analyzed the data and interpreted the results. All the authors discussed the results and revised the manuscript.

## Conflict of Interest

The authors declare that the research was conducted in the absence of any commercial or financial relationships that could be construed as a potential conflict of interest.
